# Utilising malariometric data in Real Time: a strategy to roll back malaria and sustain local elimination

**DOI:** 10.1186/1475-2875-11-S1-O29

**Published:** 2012-10-15

**Authors:** Clive Shiff

**Affiliations:** 1 Malaria Research Institute, Johns Hopkins Bloomberg School of Public Health, USA

## 

Current scale up strategies for malaria control are based on massive input of resources as well as expertise from major donors from a variety of sources but long term sustainability is insecure. Objectives are not always based on public health principles, and outcomes are measured on an immediate scale of demographic or logistic estimates rather than epidemiological indices. If the objective is to reduce transmission as well as to reduce mortality and disease in endemic communities, new strategies sustained through local infrastructure and expertise must be developed. There is a need to focus on empowering local infrastructure to collect data from rural areas, process them in real time, analyze for outbreak conditions and mobilize the local health system to intervene strategically in place and time. In Zambia, the rural health service is doing this on a pilot scale.

In endemic areas where transmission is less than holoendemic (or stable), transmission is seasonal with peaks during the rainy season and troughs when the weather is cold or hot and dry. During the peak malaria season malaria is widespread, but during the low transmission season malaria can be quite restricted and focal with a high proportion of asymptomatic infections. If a major vector control intervention is implemented, then transmission becomes reduced both during the peak but also during the dry season. This has been show clearly in the Macha district of Zambia where data on malaria case incidence rates has been collected weekly since August 2008. Staff at 14 rural health centres (RHC) report weekly by SMS texting to the research institute at Macha the number of cases diagnosed positive by rapid diagnostic test (RDT) during the previous week. During the low transmission season, it becomes clear just where residual foci occur and what appear to be salient risk factors for low season transmission. It is also clear how and when the case positivity rate spreads after the advent of the rains. Using GIS it is clear where the foci persist and where the parasite reservoir would be vulnerable to attack. These foci were identified by following cases diagnosed in RHC during the low transmission period in 2010 to their homestead, and by examining other residents of the homestead by RDT and PCR for asymptomatic cases. There were significantly more malaria infections detected at these homesteads than in homes selected randomly. We propose to target such foci in real time using RHC staff on bicycle during low transmission and treat all contacts with regular anti malaria treatment without additional diagnosis. This will concentrate the attack in place and time. With preparation, this paradigm can be integrated into the health system, would be locally sustained and in time will eliminate foci and the parasite reservoir and reduce dependence on major donations from other countries and donors whose priorities may change.

